# Does your neighborhood protect you from being depressed? A study on social trust and depression in Indonesia

**DOI:** 10.1186/s12889-019-7657-5

**Published:** 2019-10-25

**Authors:** Mohamad Fahmi, Nur Afni Panjaitan, Ibnu Habibie, Adiatma Y. M. Siregar, Gilang Amarullah, Rahma, Deni K. Sunjaya

**Affiliations:** 1 Center for Economics and Development Studies, Department of Economics, Faculty of Economics and Business, Universitas Padjadjaran, Bandung, West Java Indonesia; 2 Department of Public Health, Faculty of Medicine, Universitas Padjadjaran, Bandung, West Java Indonesia

**Keywords:** Social trust, Depression, Multilevel regression, Rasch model

## Abstract

**Background:**

Prevalence of depression in Indonesia is estimated at about 3.7% of the total population, although the actual may be higher. Studies worldwide have linked the environment where people live to their mental health status. However, little research is found in Indonesia regarding this link. We examined the association between individuals’ perception towards their neighborhood and their depression symptoms.

**Methods:**

Social trust was measured at the individual (level 1) and community (level 2) levels based on the Indonesian Family Life Survey 5 (IFLS5) in 2014. Depression was measured using the 10-item Center for Epidemiologic Studies-Depression Scale Revised (CESD-R-10) and the scores were transformed into logit form using the Rasch model. Multilevel regression was used to determine correlations.

**Results:**

Of the total sample of 14,227 respondents in this study, about 19.4% had experienced severe depression symptoms in the past week. Social trust was found to be significantly associated with severe depression symptoms. The weaker the individuals’ social trust towards their neighbourhood, the higher the probability of experiencing severe depression symptoms would be.

**Conclusions:**

This study shows that social trust is associated with the severity of depression symptoms: the higher the social trust, the lower the probability of having severe depression symptoms is. Depression symptoms may also be attributed to significant differences between communities.

## Background

Depression is a significant global issue in public health and one of the main causes of disability [1]. Depression prevalence in Indonesia has reached 3.7% of total population or about 9 million people [2] although an estimation using Indonesian Family Life Survey (IFLS) 5 in 2014 suggested that depression may reach as high as 21.8% among adults [3]. The Indonesian Ministry of Health reported that in 2013 about 14 million individuals aged 15 and above had experienced emotional mental disorders, including depression and anxiety, with more than 400,000 had suffered from severe mental disorders [3].

Depression arises as a complex interaction between social, psychological, and biological factors. A study about patients’ perception on the causes of depression stated that there are six factors contribute to depression, namely social structure deprivations, interpersonal difficulties, traumatic experiences, affective deprivations, negative self-image, and interpersonal loss [4]. Social capital has been reported to explain many variations in the health status of communities and societies including the mental health status [4]. Several studies have also examined associations between social capital and depression in various settings such as among older populations [5, 6], fragile families [7], and pregnant mothers [8].

Social trust is identified as a cognitive social capital which refers to what people feel. It comprises of individual and generalized/community trust [9] and is a core component of social capital that is often used as the key or best indicator [10]. For instance, a study in the UK has shown that generalized trust was the only social capital variable that has a positive and highly significant association with psychological health overtime [11]. Trust as a part of social capital also has a significant relation with self-rated health in South Africa [12].

Depression has also been linked to the sub-dimensions of social capital e.g. structural (i.e. “extent and intensity of associational links or activity”) and cognitive components (i.e. “perceptions of support, reciprocity, sharing and trust”) [13, 14], social trust [15], and social participation [16]. Low “interpersonal trust” is found as an independent risk factor for “new-onset and long-term depression” [17]. Depression was found negatively associated with social trust. A study in South Africa found that the higher the individuals’ trust in their neighbourhood, the lower the probability of them to suffer from depression will be [18]. Similar result was found in the United States which concluded that social trust is negatively associated with major depression [14]. However, another study in South Africa has shown that high social trust is associated with increased depression symptoms if the district level trust is low and vice versa [19], which indicates the importance of community role in determining the relation between social trust and depression.

Despite the extensive studies published on social trust and depression worldwide, such studies are limited in Indonesia. Tampubolon and Hanandita [20] stated that individual social trust has a positive association with mental health. A few other studies in Indonesia show that trust as a part of social capital is negatively correlated with mental illness status and depressive symptoms among adults, women and older individuals [3, 21, 22]. Miller et al. [23] found a positive empirical association between participation in community and good health. For mental health, they implemented indicators of four categories, i.e. sadness, anxious, insomnia, and short temper. The existence of a family planning group was correlated/associated with less sadness and less insomnia. A pharmacy garden was also correlated with less insomnia. For insomnia, improved health was significantly correlated with the pharmacy garden (i.e. a community garden, where members will have their own plot, usually for cultivating traditional medicine) and the family planning group. For short temper, better outcomes are strongly and significantly associated with the pharmacy garden.

Lack of evidence of social trust and depression is critical in Indonesia since low levels of social trust could lead to the increase of depression prevalence. The aim of this study was to determine whether social trust at the individual and community levels were associated with individual depression symptoms using a multilevel analysis. To our knowledge, this study is among the first, if any, that shows such association. This could have important public health implications as it can serve as an evidence to support the potential of social trust in preventing depression.

## Methods

We used data from the Indonesia Family Life Survey (IFLS), which is an on-going longitudinal survey that focuses on measuring the socioeconomic and health status of the country. The IFLS sample was representative of about 83% of the Indonesian population and contained over 50,000 individuals living in 13 provinces in the country [24]. IFLS focuses on collecting data of individual respondents, their families, their households, the communities in which they live, and health and education facilities they use. The database also includes various indicators of social trust and mental health. IFLS5 data was conducted in 2014–2015, consists of data from interviews with 16,204 households and 50,148 individuals. Specific for mental health and trust modules the sample size are 31,447 and 31,595 individuals, respectively, due to missing data. Finally, after data cleaning, 14,227 individuals were used in our regression analyses.

### Measurement of depression

IFLS5 used the CESD-R-10 scale to identify symptoms of depression. The scale consists of 10 items that address how the individual felt in the past week. Eight items cover negative feelings, such as *“I was bothered by things that usually don’t bother me*”, and the remaining two items covers positive feelings, such as “*I was happy*”. Respondents rated their feelings from 1 to 4, where 1 = Rarely or never (≤1 day); 2 = Some days (1–2 days); 3 = Occasionally (3–4 days); and 4 = Most of the time (5–7 days). This scale is modified from the standard CESD-R-10 scale, which uses a rating system that started with a score of 0; thus, Rarely or never (≤1 day) was given a score of 0 and the scale ranged from 0 to 3 with a maximum score of 30. In that system, individuals with a score of 10 or more are considered depressed [25]. Note that this cut off has not been validated for Indonesia. For this study, we used the Rasch model to convert the scores from the standard questionnaire to values that could be evaluated in a logarithmic form (i.e., range: 1 to 4) using Winstep 3.73.0 software.

The CESD-R-10 scale has been used widely as a tool for measuring depression symptoms. The scale is found to be valid and reliable in identifying individuals at high risk of developing major depression in clinical populations. Nevertheless, its structural validity has been challenged (Cole et al., 2004 as cited in Covic [26]). The Rasch model analysis can be applied to resolve the known impediments [26] and, as Wright & Mok [27] explained, it also fulfils the five requirements for a good tool to validate an instrument. These requirements are as follows: (a) produce linear measures, (b) overcome missing data, (c) give estimates of precision, (d) have devices for detecting misfit, and (e) the parameters of the object being measured and of the measurement instrument must be separable. It is argued that only the family of Rasch measurement models are able to address these problems.

Rasch model has various techniques for different purposes. The first model introduced was a simple model for responses to dichotomous items which was used in educational test by Georg Rasch in 1960. Since then, the model has developed rapidly from its original model (Ordered Polytomous Items by Andrich in 1978, followed by partial models by Masters in 1982, to facests models by Linacre in 2004 [28]. Since responses of CESD-R-10 is in an ordinal form without a clear interval, as criticized by Kuzon et al. [29], they cannot be used in parametric calculations. Consequently, we used the Rasch measurement of Ordered Polytomous Items for this study in order to convert the polytomous scores from the standard questionnaire into values that could be evaluated in a logarithmic form (i.e., range: 1 to 4).

### Measurement of social trust

IFLS5 has specific questions to examine respondents’ trust of neighbors. These start with general questions on trusting other people in the village go on to specific questions about whether respondents would leave their children with their neighbors.

In this study, social trust was measured by individual-level and community-level predictor variables. To evaluate individual social trust, we used all seven indicators of trust in IFLS5, in line with a study by Tampubolon & Hanandita [20]. These indicators referred to respondent’s trust of neighbors. These start with general questions about trusting other people in the village, and go onto ask specific ones, such as whether respondents would leave their children with their neighbors. In particular, participants responded to the following seven items: (1) I am willing to help people in this village, if they need it; (2) In this village, I must be alert, because people are likely to take advantage of me; (3) Taking into account the diversity of ethnicities in the village, I place more trust in people of my own ethnicity; (4) I would be willing to leave my children with my neighbors for a few hours, when I cannot take my children with me; (5) How safe do you consider this village? (6) In most parts of the village, is it safe for you to walk alone at night? and (7) I would be willing to ask my neighbors to look after my house if I left for a few days. The responses to questions 1–4 and 7 included strongly agree, agree, disagree, and strongly disagree with 1 for strongly agree and 4 for strongly disagree. The responses to questions 5 and 6 included very safe, safe, unsafe, and very unsafe with 1 for very safe and 4 for very unsafe. We reverse coded questions 2 and 3 so that they have similar pattern of responses as with the other questions. We used the Rasch model to convert the responses into a single measurement of trust in a logarithmic form. After we convert the score into logarithmic form, we reversed the score by multiplying the score in logarithmic form by − 1 so that lower score represents lower individual level social trust and higher score represents higher individual level social trust. The final score ranged from − 4.51 to 4.16. The score that was closer to − 4.51 represented lower individual level social trust while the score that was closer to 4.16 represented higher individual social trust.

The similar items were also asked to community leaders and we used their responses to construct the community social trust variables. We opted to use community leader response for measuring community-level social trust based on a perception that community leaders have extensive knowledge and understanding about their community [30]. In addition, community leaders can stimulate social capital by nurturing collective action through coordination and conflict resolution for mutual benefit [31]. Several past researches have noted limitations in forming neighborhood-level measures based on aggregate individual perceptions. Aggregating individual responses of residents living in communities characterized by high levels of mobility may result in neighbourhood measures exhibiting poor internal consistency [32]. Community leaders responded to the following seven items: (1) People in this village are always looking out for each other; (2) Most people in the village are willing to help if you need it; (3) In this village one has to be alert or someone is likely to take advantage of you; (4) In this village, residents from the same ethnicity trust each other more than they trust those with different ethnicity; (5) In this village, residents from the same religion trust each other more than they trust those with different religion; (6) Would people in this village be willing to leave their children with their neighbors for a few hours if they cannot bring their children with them?; (7) Would people in this village be willing to ask their neighbors to look after their house if they leave for a few days? We reverse coded questions 3, 4, and 5 so that they have similar pattern of responses as with the other questions. As with the individual social trust, we used the Rasch model to convert the responses into a single measurement of trust in a logarithmic form and later reversed the score by multiplying the score in logarithmic form by − 1 so that lower score represents lower community level social trust and higher score represents lower community level social trust. The final score ranged from − 2.3 to 4.9. The score that was closer to − 2.3 represented lower community level social trust while the score closer to 4.9 represented higher community social trust.

### Covariates

This study included the following covariates: age of the respondent at the interview, gender, marital status, employment status, education, per capita household expenditure, and location (urban/rural). Age was treated as a continuous covariate. Gender was entered as a dummy variable (1: male; 0: female) while marital status was entered using not married as the reference group. Employment status was measured with a dummy variable (1: working; 0: otherwise) while for education we included three dummies variables, namely junior high school (1: completed junior high school; 0: otherwise), senior high school (1: completed senior high school; 0: otherwise), and universities (1, completed universities school; 0: otherwise). The per capita household expenditure variable was entered as a continuous variable (in logarithmic scale). Location is a dummy variable which indicates whether the individual resides in urban areas or not with rural areas as the reference.

### Multilevel regression

We applied a multilevel mixed-effect linear regression analysis, which included factors that influenced variations in mental health outcomes, including compositional factors (e.g. age, sex, educational attainment, income) and contextual factors (i.e. community social trust). Multilevel regression can take into account the clustered structure of the data allowing for residual components at each level in the hierarchy. In this study, a two-level model allowed for grouping of individual outcomes within neighborhood/community and would include residuals at the individual and neighborhood/community. Thus, the residual variance is partitioned into the between neighborhood/community component (the variance of the neighborhood/community-level residuals) and the within neighborhood/community component (the variance of the individual-level residuals). We conducted five regression analyses: 1) null model (regressed only using community ID); 2) regressed against community social trust and community ID; 3) regressed against individual social trust and community ID; 4) regressed against community and individual social trust and community ID; and 5) regressed against all variables (including covariates). We did not include weights into our analysis. We used Stata 13 software for conducting our regression analysis.

## Results

### Descriptive statistics

We included 14,227 respondents in the analysis. The depression scale was transformed from the CESD-R scores to the logit form using the Rasch model (Table 1). The depression scores ranged from − 3.5 to 2.0 with individuals who were experiencing depression symptoms would have a score that was close to 2.0 while individuals who did not experience the symptoms would have a score that was close to − 3.5. A CESD-R original score of larger than 10 indicates severe depression symptoms [25], which is similar to − 0.52 in logit form. Thus, we considered respondents with higher than − 0.52 to have severe depression symptoms.

**Table 1:** Depression, social trust, and socioeconomic characteristics of 14,227 participants in the IFLS5

Variable	Mean or %	Std. Dev.	Min	Max
Depression score (*n* = 14,227)	−1.2	0.9	−3.5	2.0
Social Trust scores: individual-level	− 0.19	0.85	−4.51	4.16
Social Trust scores: community-level	0.53	0.99	−2.3	4.9
Age (year)	40.2	14.1	15.0	94.0
Male (male = 1, female = 0)	40%			
Marital Status (married = 1, not married = 0)	83%			
Employment Status (working = 1, not working = 0)	70%			
Smoking status (smoking = 1, not smoking = 0)	40%			
Log per capita expenditure (*n* = 13,443)	13.6	0.6	11.2	16.9
Place of stay (urban = 1, rural = 0)	60%			
Last attained education level				
Junior High	20%			
Senior High	30%			
University	5%			
(primary school and not going to school as reference)				

The socioeconomic characteristics of the respondents are shown in Table 1. Social trust score of respondents ranged from − 4.51 to 4.16 with a mean value of − 0.19. The average age of the respondents was 40.2 years. The youngest respondents were 15 years old, and about 15% of respondents were above age 55 years. About 83.6% of respondents were married, 70.5% were working, and 37% had smoking habits. About 52.9% of respondents completed junior high school education and the remainder had attained primary school education or less.

### Regression analysis

The multilevel regression analysis showed a significant negative association between social trust at the individual level and severe depression symptoms. A higher social trust score is associated with lower score in depression symptoms (Table 2). This finding is consistent with the study of Fujiwara and Kawachi [14] in the United States which found that those who trust their neighbours are less likely to develop major depression.

**Table 2:** Multilevel regression results

	(1)	(2)	(3)	(4)	(5)
Variables	Depression	Depression	Depression	Depression	Depression
Community Social Trust		−0.007(− 0.48)		− 0.006(− 0.41)	−0.007(− 0.49)
Individual Social Trust			−0.054***(−6.25)	−0.054***(−6.25)	− 0.041***(−4.51)
Age					−0.006***(−10.60)
Male (male = 1, female = 0)					−0.049(−1.95)
Marital Status (married = 1, not married = 0)					−0.157***(−7.53)
Employment Status (working = 1, not working = 0)					0.004 (0.21)
Smoking Status (smoking = 1, not smoking = 0)					0.092***(3.66)
Log Per capita expenditure					−0.038**(−2.79)
Place of stay (urban = 1, rural = 0)					0.070* 2.26)
Junior High (completed junior high = 1, 0 = otherwise)					−0.067**(−3.04)
Senior High (completed senior high = 1, 0 = otherwise)					−0.073***(−3.44)
University (completed university = 1, 0 = otherwise)					−0.195***(−4.88)
Constant	−1.206***(−79.54)	−1.203***(−70.81)	−1.216***(−79.48)	−1.213***(−70.82)	−0.314 (−1.72)
Var (cons)	−1.512***(−26.59)	−1.512***(−26.60)	− 1.506***(− 26.63)	−1.506***(− 26.64)	− 1.556*** (−26.39)
Var (Residual)	−0.133***(−22.19)	−0.133***	− 0.134***(−22.19)	−0.134***(− 22.43)	− 0.144***(−23.38)
Intra-class correlation (ICC)	.0596	.0596	.0604	.0604	.0561
Akaike Information Criterion (AIC)	36,989.95	36,991.72	36,952.91	36,954.74	34,664.91
N	14,227	14,227	14,227	14,227	13,442

*t* statistics in parentheses

^*^ *p* < 0.05, ^**^ *p* < 0.01, ^***^ *p* < 0.001

This table shows the multi-level mixed effects linear regression results

The intraclass correlation (ICC) for model 1 (null model) was 6%, meaning that 6% of the variation in depression score was due to variation between communities. However, the community social trust variable itself was not significant although the variable sign showed that better community social trust is associated with a smaller number of individuals with severe depression symptoms. The variance inflation factor (VIF) for all independent variables is presented in Additional file 2.

Our covariate analysis showed that age was significantly negatively related to depression. This finding indicated that older individuals had a lower probability of experiencing depression. Marital status was also related to depression. An unmarried, divorced, or separated status was associated with a higher risk of experiencing depression. Smoking was also associated with a higher probability of experiencing depression. Junior high, secondary, and university educational levels were negatively associated with depression; thus, the higher the educational level, the lower the probability of experiencing depression will be.

We ran the full model stratified by gender, and found that the variable signs to be similar with our original regression analysis, although the significance of a few variables differs, as with the case of regression analysis among male respondents (Additional file 1). This shows that in general our results persist within different gender group.

## Discussion

This study shows that social trust, which is represented by the individuals’ perception of trust towards their neighbourhood, is negatively associated with depression symptoms. Our multilevel regression analysis indicates that, although statistically insignificant, a community may to some extent associated with lower incidence of depression and that the depression incidence may vary between communities. This implied that the community we live in plays a significant role as a factor influencing depression symptoms. Studies in Indonesia show that trust as a part of social capital is negatively associated with mental illness status and depressive symptoms among adults, women and older individuals [3, 21, 22]. The results of this study are consistent with the findings from global studies [5, 18, 33–35] that social trust, neighborhood preference, sense of belonging, perception of trust, perception of solidarity and friendliness, perception of reciprocity, and safety are all associated with better mental health status and fewer depression symptoms. Thus, it seems that fostering conducive and safe communities to reduce the incidence of depression is has a potential to prevent the disease.

The use of social trust as a prevention measure for mental illness has been recognized. In Japan, neighborhood bonding is estimated to be a potential prevention against depressive mood in old age [36], while social trust in a neighborhood is associated with lower infant physical abuse [37], a potential source for depressive disorder [38, 39]. In Korea, trust among family members may lead to better depression outcome [40]. Potentially, prevention efforts that promote social trust can be applied in Indonesia as well. Creating active community/neighborhood and trust in Indonesia should be feasible given its *gotong royong* value (sharing burdens, mutual cooperation, mutual assistance) [41], and most notably in the rural/urban village areas [42]. Indeed, it has been suggested that community participation in Indonesia may lead to a better mental health state [21, 22]. Hence, the value of *gotong royong*, if correctly implemented, may potentially reduce the probability of depressive disorders to occur from causes such as old age, child abuse, and lack of trust within family or household.

However, utilizing social trust as prevention for depressive symptoms has its own challenges. Currently, Indonesia is facing rising and stagnant inequality [43, 44]. Income inequality is a potential factor that may lead to negative effects on wellbeing and/or life satisfaction which then erodes social trust [45–47] while social deprivation may lead to depression [48]. Furthermore, for households with lower socio-economic status, community involvement is also low [49], which is a potential cause for low social trust and depression. In our result, expenditure per capita has a negative correlation with the onset of depression symptoms. Given these findings, it is also possible that *gotong royong* value will be harder to apply in the settings where income inequality and social deprivation presents because the level of trust is already potentially low in these settings.

Another potential challenge is ethnic diversity, which is the case in Indonesia. The huge ethnic diversity in Indonesia results in an increase in tolerance, but a decrease in trust, perceived safety, and community participation [50]. In Denmark, such condition also occurs in which more diversity decreases trust [51]. Ethnic diversity may become a larger issue in Indonesia when it is related to tribal nationalism given the existence of social media where freedom of speech and hate occur at the same time [52]. In addition, in the case of ethnic minority, subsequent discrimination will most likely relate to the severity of psychotic and depression symptoms [53]. As such, recognizing ethnic diversity as both an opportunity and challenge to build social trust is an important aspect, especially in the case of Indonesia that has more than 600 and 20 tribes and languages, respectively [54].

Hence, in the context of Indonesia where income inequality, social deprivation, ethnic diversity, and tension are still major issues, albeit with a strong value of mutual cooperation, a unique intervention to promote social trust is needed. Creating interventions promoting only active community engagement will not be sufficient without incorporating specific condition of every region, especially in terms of income inequality, social deprivation, ethnic diversity, and the sense of mutual cooperation. Interventions promoting social trust should take these factors into account to achieve the highest probability to prevent depression effectively.

This study has several weaknesses. First, CESD-R-10 defines depression as an episode of depression symptoms that lasted for seven days. Despite the wide use of this tool, this definition is limited because the diagnosis of depression requires that the symptoms must be present for at least two weeks [55]. Thus, the short duration of depression symptoms presented in CESD-R 10 might lead to potential weakness of the instrument. However, studies that have used this instrument [17, 20] show that this weakness does not necessarily cause the instrument to be unusable as it still generates reliable results.

Second, the CESD-R 10 version used on IFLS data are not yet formally validated for Indonesia, although the Survey Meter, which is the research firm that collects IFLS data, stated that they have conducted extensive tests. Consequently, the version used in IFLS may potentially have lower accuracy than the validated version of CESD-R. The validation process for Indonesia is currently ongoing [56] and is currently in the process of report writing. Using this version for future data collection may generate more reliable results.

Third, we only used one IFLS wave, thus we derive our conclusion from cross sectional data. As such, we are unable to show whether the respondents with severe depression in our study is actually caused by the low level of social trust and vice versa. Our analysis is merely showing the existing association between depression symptoms and social trust.

Fourth, we did not include weights in our data analysis so our results may not necessarily representative of the whole population of Indonesia. However, given the relatively large sample used in our analysis, we believe it has been able to produce a result that can reflect the situation in Indonesia to some extent, especially regarding the relationship between social trust and depression symptoms. Furthermore, since after data cleaning we missed almost 20,000 data points, there may be a risk of selection bias, although this requires further inspection or study. However, readers should consider this aspect when they interpret our results.

Lastly, although we have tried to include confounding factors into our analysis, it is most likely that there are variables that we missed. For instance, although we only include income inequality, social deprivation, and ethnic diversity as potential challenges to promote social trust as depression prevention, including these factors as control variables may lead to a better estimation.

## Conclusions

This study aimed to determine whether social trust at the individual and community levels was associated with the probability of having more depression symptoms. With a multilevel analysis, we found that individual and community trust has a negative relation with severe depression symptoms. Interventions to improve social trust may lead to less people with severe depression symptoms; nevertheless, factors such as income inequality, social deprivation, ethnic diversity and tension, and the sense of mutual cooperation should be taken into account to increase the effectiveness of the interventions.

## Supplementary information

**Additional file 1.** Multilevel regression results for male and female.

**Additional file 2.** Variance Inflation Factor (VIF) result.

## Data Availability

The datasets generated and/or analyzed during the current study are freely available in the RAND repository, https://www.rand.org/labor/FLS/IFLS.html

## Abbreviations

CESD-R-10: the 10-item Center for Epidemiologic Studies-Depression Scale Revised
IFLS4: Indonesia Family Life Survey 4
IFLS5: Indonesian Family Life Survey 5
VPC: Variance partition coefficient
WHO: World Health Organization
