# Supplementary material for: Does your neighborhood protect you from being depressed? A study on social trust and depression in Indonesia
Source: BMC Public Health. 2019 Oct 25;19:1371. doi: 10.1186/s12889-019-7657-5 (PMC6814976; doi:10.1186/s12889-019-7657-5)
Supplement: Supplementary file 1 — Additional file 1. Multilevel regression results for male and female. [file 12889_2019_7657_MOESM1_ESM.rtf]

Additional Files A.
Multilevel regression results for male and female
	(1)	(2)	
	Male	Female	
Community Social Trust	-0.00203	-0.00862	
	(-0.12)	(-0.50)	
Individual Social Trust	-0.0198	-0.0579***	
	(-1.46)	(-4.68)	
Age	-0.00719***	-0.00605***	
	(-7.89)	(-7.02)	
Marital Status	-0.201***	-0.115***	
	(-5.54)	(-4.23)	
Working Status	-0.0592	0.0254	
	(-1.61)	(1.19)	
Smoking Status	0.0799**	0.215***	
	(2.82)	(3.72)	
Log Per capita expenditure	-0.0662***	-0.0127	
	(-3.33)	(-0.71)	
Urban	0.0848*	0.0624	
	(2.40)	(1.77)	
Junior High	-0.0719*	-0.0700*	
	(-2.20)	(-2.36)	
Senior High	-0.0398	-0.115***	
	(-1.33)	(-3.91)	
university	-0.215***	-0.188***	
	(-3.72)	(-3.41)	
Constant	0.144	-0.707**	
	(0.53)	(-2.91)	
Observations	5973	7469	
			
t statistics in parentheses
* p < 0.05, ** p < 0.01, *** p < 0.001


This table shows the multi-level mixed effects linear regression results for both male and female samples.
