# Supplementary material for: Does your neighborhood protect you from being depressed? A study on social trust and depression in Indonesia
Source: BMC Public Health. 2019 Oct 25;19:1371. doi: 10.1186/s12889-019-7657-5 (PMC6814976; doi:10.1186/s12889-019-7657-5)
Supplement: Supplementary file 2 — Additional file 2. Variance Inflation Factor (VIF) result. [file 12889_2019_7657_MOESM2_ESM.docx]

| **Variable** | **VIF** | **1/VIF** |
| --- | --- | --- |
| Male (male = 1, female = 0) | 2.70 | 0.37 |
| Smoking status (smoking = 1, not smoking = 0) | 2.59 | 0.39 |
| Age (year) | 1.22 | 0.82 |
| Senior High (completed senior high=1, 0=otherwise) | 1.39 | 0.72 |
| Junior High (completed junior high=1, 0=otherwise) | 1.31 | 0.76 |
| University (completed university=1, 0=otherwise) | 1.14 | 0.88 |
| Employment Status (working = 1, not working = 0) | 1.17 | 0.85 |
| Log per capita expenditure | 1.10 | 0.91 |
| Place of stay (urban = 1, rural = 0) | 1.08 | 0.93 |
| Individual Social Trust | 1.06 | 0.94 |
| Marital Status (married = 1, not married = 0) | 1.03 | 0.97 |
| Community Social Trust | 1.00 | 0.99 |
| **Mean VIF** | **1.40** |  |

Additional files B. Variance Inflation Factor (VIF) result

This table provides the VIF result of the independent variables used in the multi-level mixed effects linear regression
